# Prediction of Compressive Strength of Partially Saturated Concrete Using Machine Learning Methods

**DOI:** 10.3390/ma15051662

**Published:** 2022-02-23

**Authors:** Ma. Doreen Esplana Candelaria, Seong-Hoon Kee, Kang-Seok Lee

**Affiliations:** 1 Department of ICT Integrated Ocean Smart Cities Engineering, Dong-A University, Busan 49315, Korea; mecandelaria@up.edu.ph; 2 Institute of Civil Engineering, University of the Philippines Diliman, Quezon City 1101, Philippines; 3 Department of Architectural and Smart Cities Engineering, Hanyang University, Ansan 15588, Korea; ksleenist@hanyang.ac.kr

**Keywords:** data fusion, ultrasonic pulse velocity, electrical resistivity, marine environment, compressive strength

## Abstract

The aim of this research is to recommend a set of criteria for estimating the compressive strength of concrete under marine environment with various saturation and salinity conditions. Cylindrical specimens from three different design mixtures are used as concrete samples. The specimens are subjected to different saturation levels (oven-dry, saturated-surface dry and three partially dry conditions: 25%, 50% and 75%) on water and water–NaCl solutions. Three parameters (P- and S-wave velocities and electrical resistivity) of concrete are measured using two NDT equipment in the laboratory while two parameters (density and water-to-binder ratio) are obtained from the design documents of the concrete cylinders. Three different machine learning methods, which include, artificial neural network (ANN), support vector machine (SVM) and Gaussian process regression (GPR), are used to obtain multivariate prediction models for compressive strength from multiple parameters. Based on the R-squared value, ANN results in the highest accuracy of estimation while GPR gives the lowest root-mean-squared error (RMSE). Considering both the data analysis and practicality of the method, the prediction model based on two NDE parameters (P-wave velocity measurement and electrical resistivity) and one design parameter (water-to-binder ratio) is recommended for assessing compressive strength under marine environment.

## 1. Introduction

Concrete in buildings and civil infrastructure systems is employed to mainly resist compressive stress in structural members under various external loadings. Consequently, compressive strength of concrete, *f_c_*, is one of the most critical engineering parameters describing performance of concrete. In many general design codes [1,2], compressive strength of concrete is used to estimate the other important engineering properties for strength and deformability of concrete (e.g., tensile strength, shear strength, debonding strength and elastic modulus of concrete). Compressive strength of concrete is a fundamental parameter for elastic and plastic analyses of reinforced concrete structures for designing new structures and/or evaluation of structural integrity of old/exiting structures. Measurement of *f_c_* is needed in the new construction sites to determine the timing of critical site works such as reshoring, demolding and post-tensioning and to achieve quality control and quality assurance (QA/QC) [3,4,5]. It is reported that *f_c_* has a good correlation with durability indices (e.g., chloride permeability and coefficient of chloride diffusion) [6,7,8]. Therefore, it is important to evaluate *f_c_* in structures to better understand the structural integrity and durability of new and existing structures.

There are several measurements methods for evaluation of compressive strength of concrete in the laboratory and the filed practices. First, compressive strength of concrete can be typically assessed using a destructive method on regularly shaped concrete samples in construction sites and/or core samples directly collected from various sections of concrete members [3]. While this approach may provide precise data, it is time-consuming, costly and may cause additional damage to the structure being investigated. Furthermore, to achieve reliable results, many concrete cores will need to be obtained across the entire concrete structure, which will significantly increase cost and effort and restrict the use in field practice. For structural health monitoring of concrete infrastructures, several nondestructive evaluation (NDE) techniques are now in use. One of the popular methods used for NDE assessment is the measurement of ultrasonic pulse velocity (UPV) [1,2,9,10]. Measurement of UPV requires a simple process and the method is already standardized in many countries. Estimation of concrete’s compressive strength has been done several times. The study by Hong et al. [11] correlated the UPV with the compressive strength of concrete according to the age. This way, compressive strength of concrete at early stage can be estimated by UPV that may help QA/QC of concrete during construction. In the study of Saint-Pierre et al. [12], UPV is used to designate concrete quality in old concrete structures. Omer et al. [13] proposed a relationship between compressive strength of concrete and UPV of ground granulated ballast furnace slag (GGBFS)-based geopolymer mortars that were exposed to elevated temperatures. The study of Owaid et al. [14] correlated the UPV with the strength of thermally activated alum sludge (AAS) multiple blended high-performance concretes. Their study concluded that UPV values are affected by the pozzolanic materials and the AAS. More recently, Presa et al. [15] studied the relationship between *f_c_* and UPV in samples of mortars with 25% of pozzolanic content.

Until now, P-wave velocity calculations have been mostly used in the laboratory and in the field practice to assess UPV of concrete and to estimate *f_c_*. However, P-wave velocity of concrete is affected by many factors such as smoothness of contact surface during test, temperature, moisture content, material and mix proportion and presence of reinforcing steel or fiber [16,17] that is not directly correlated with compressive strength of concrete values. It may not be enough to estimate the mechanical properties of concrete as was shown in a previous study [18,19,20]. Recently, some researchers demonstrated that the use of S-wave velocity can be used for estimating concrete properties because it has a strong association with concrete mechanical properties, with less susceptibility to other material and environmental impacts [21,22,23]. However, there are still practical and theoretical limitations of using S-wave velocity measurements in the field practice. For examples, generation of pure S-wave is a challenging task compared to the use of P-wave, and compared to P-wave, experimental data on the S-wave velocity and compressive strength is limited in the literature. In summary, it is not sufficient to solely use an UPV parameter (P-wave or S-wave) for reliable and consistent prediction of compressive strength of concrete under various environmental conditions.

Some researchers argued the importance of the use of multiple NDE parameters that complement the use of UPV. Each individual nondestructive test (NDT) evaluates a different set of parameters such as UPV for wave velocities [24,25,26], rebound hammer for hardness [27,28,29], ground penetrating radar for relative permittivity [30,31] and electrical resistivity (ER) measurements for electrical resistivity [32,33,34]. It has been demonstrated that the use of combined parameters, using UPV as one of the parameters, is effective for a more reliable evaluation or estimation of compressive strength of concrete [35,36,37,38,39,40,41,42,43,44]. These studies paired UPV with other parameters such as weight of concrete [35,37], properties of concrete aggregate [36,43], water-to-cement ratio, fly ash content, humidity, age and micro-silica [38,41,43]. Some researchers combined UPV with other nondestructive test parameters from electrical resistivity, ground penetrating radar [39] and rebound hammer [44].

There have been several data fusion methods that relate multiple sensor data to estimate compressive strength of concrete. Regression analysis is one of the common methods that has been used for estimation of mechanical properties of concrete, specifically, its compressive strength. For predicting associated tasks, regression techniques are the simplest and most efficient; however, the performance of the regression analysis results is strongly dependent on a relationship between various parameters (independent parameters) and compressive strength of concrete (dependent variable) that is predefined before the regression analysis. Therefore, it is necessary to establish consolidated knowledge background on the variables of interest in regression techniques. Another method of data fusion is through machine learning. Machine learning algorithms employ computational methods to “learn” information directly from data rather than relying on a model based on a predetermined equation. As the number of samples available for learning grows, the algorithms adjust their performance. Examples of this methods are the artificial neural network (ANN), support vector machines (SVM) and Gaussian process regression (GPR). Most studies that use regression analysis for prediction of compressive strength of concrete compare the results with ANN analysis [35,36,38,39,41,42,45]. Other studies have also used ANN in improving the measurement accuracy of some equipment like flowmeter in measuring gas volumetric percentage [46,47]. Some studies on prediction of concrete compressive strength have used SVM in regression [48,49,50,51,52,53,54]. SVM is a supervised learning technique for solving classification and regression problems with data. An SVM training algorithm creates a model that assigns new examples to one of two categories using a set of training patterns that are each labeled as belonging to one of two categories. In addition, the nonparametric, Bayesian approach to regression known as GPR is making waves in the field of machine learning. GPR has many advantages, including the ability to work with small datasets and provide uncertainty measurements on forecasts. Because GPR is nonparametric (i.e., not constrained by a functional form), rather than computing the probability distribution of parameters of a single function, GPR computes the probability distribution of all admissible functions that fit the data. Few studies have already used GPR in estimating properties of concrete [55,56,57,58,59,60]. Table 1 summarizes the principles, advantages and limitations of the four data fusion methods used in the prior studies.

While data fusion has been widely used in estimating the properties of concrete, it is seldom that the effect of water saturation and other environmental factors, such as presence of sodium chloride in concrete, are included in estimating the concrete compressive strength. Concrete, a porous and heterogenous material, comprises several types of voids (e.g., entrapped air voids, capillary voids, interface space in C-S-H and entrained air bubbles) [61] that can be infiltrated by other materials like water and salt. Moreover, with recent technologies in sourcing the raw materials for concrete batching, it is also important to consider the effect of other recycled compositions of the materials. Some recent studies [62,63] have investigated the effect of such materials in the performance of concrete. Water in concrete pores has been found to have a significant impact on concrete’s mechanical and durability properties [64]. It has been demonstrated that the increasing moisture content (or water saturation level) decrease the compressive strength of concrete [61,64,65,66,67]. Mechanical properties and durability of concrete are two distinct factors that can describe the quality of concrete. Several studies have been made to relate the durability and compressive strengths of concrete [68,69,70,71,72,73,74]. While durability and compressive strength are two different characteristics of concrete, they share some of the indicators. In this study, some durability factors were selected to estimate the compressive strength of concrete such as moisture content and water-to-binder ratio.

The objective of this study is to estimate the compressive strength of concrete using the combination of different NDT parameters, ultrasonic velocities (P- and S-waves) and ER of concrete, and two physical parameters of concrete, density and water-to-binder ratio of concrete. Experimental and data fusion materials and methodology of this study is discussed in the succeeding section. The concrete specimens used in the experimental part were commercially sourced from a batching plant and the details of material properties are described in Section 2. The research would evaluate the optimum combination of these five parameters to give a more reliable estimation of the compressive strength of concrete. Finally, different data fusion will be compared to determine the optimum way of combining the different parameters. For this study, MATLAB tools for neural network and regression learner would be used for data fusion analysis. With the data and results from the present study, it is anticipated that estimation of compressive strength of concrete under various environmental conditions will be improved.

## 2. Materials and Methods

### 2.1. Experimental Studies

#### 2.1.1. Sample Preparation and Water and NaCl Saturation

Sample cylinders, with 200 mm height and 100 mm diameter, were manufactured for all the tests that were done for the study. Three concrete mixes were used, with different water-to-binder ratios—MIX 1, MIX 2 and MIX 3. The properties and quantities of the samples are presented in Table 2.

**Figure 1:**
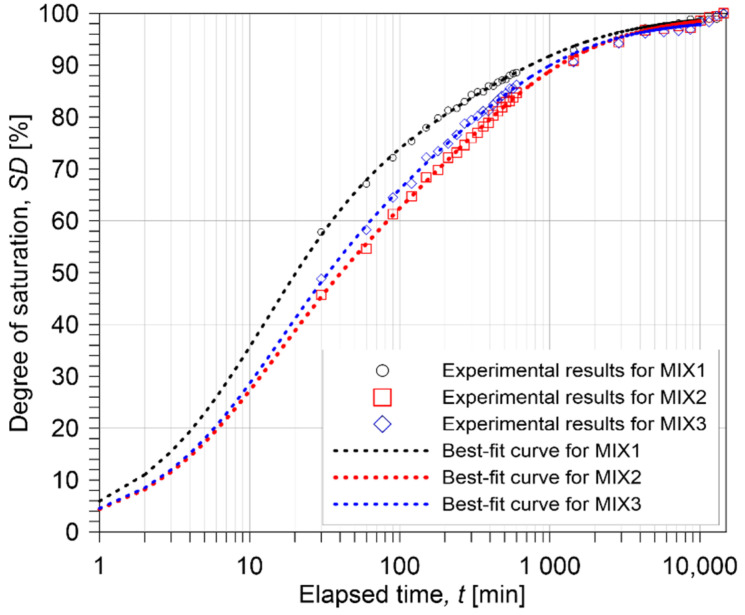
The curve of water saturation with stages in water saturation trend: variation of saturation of the three concrete types (MIX 1, 2 and 3) in linear time scale.

Saturation curves were developed to use as reference for the tests on saturated concrete cylinders, both in water and water–NaCl solution. Five target saturation levels were considered for this study—standard saturations (oven-dry and saturated-surface dry), 25%, 50% and 75%. The variables for this procedure were the proportion of the mix and the time spent immersed in the water. Three examples from each design mix were utilized in this approach, for a total of nine cylindrical specimens. After being cured in water for at least 150 days, the specimens were dried in an electronic oven (KST, Busan, South Korea) for at least 72 h at a constant temperature of 105 °C. The specimens’ mass was measured thirty minutes after they were removed from the oven. The specimens were then placed in small tanks in groups, and tap water was gently added to ensure that they remained submerged in water after the initial water absorption of the concrete. For the first ten hours, the mass of the specimens was recorded every 30 min. Excess water was wiped off the specimens with a moist cloth when they were removed from the tanks. The cylinders were then returned to the water for continuous immersion once the mass was recorded. Then, the mass was continuously measured every 24 h until the tenth day of immersion time. Figure 1 shows the saturation curves that were developed from this method. In this study, numerical formula that describes the saturation of concrete cylinders with time was determined by non-linear regression analysis of measured data based on a rational equation as follows,

(1) $$SD=\frac{\sum_{i=1}^{3}a_{i}t^{\left(i-1\right)}}{\sum_{i=1}^{2}b_{i}t^{\left(i-1\right)}}$$

where *SD* represents the estimated degree of saturation in a unit of % at time *t* after an oven-dry concrete cylinder is immersed in water, $a_{i}$ and $b_{i}$ are constant coefficients of the rational equation in Equation (1) and the subscript *i* is index of the constants. Table 3 summarizes the constant coefficients values for MIX 1, 2 and 3 concrete cylinders determined by non-linear regression analysis. Estimated saturation curves are presented as dash lines in Figure 1. In this study, the approximate time to obtain concrete cylinders with the target saturation degree (25%, 50%, 75% and 100%) was determined from the estimated saturation curves, which are summarized in Table 4. In this study, it was confirmed that estimated saturation curved for water saturation is still valid for estimating those for the NaCl saturation.

During testing, the actual saturation was an estimate of the target saturation from the reference curve. The actual readings in this study are extremely close to the target saturation levels, demonstrating the procedure’s efficiency. After actual saturation of the cylinders, nondestructive and uniaxial compressive tests were performed. The inclusion of saturating concrete specimens with NaCl was studied to consider the effect of other environmental factors in the properties of concrete.

#### 2.1.2. Ultrasonic Pulse Velocity Measurement

**Figure 2:**
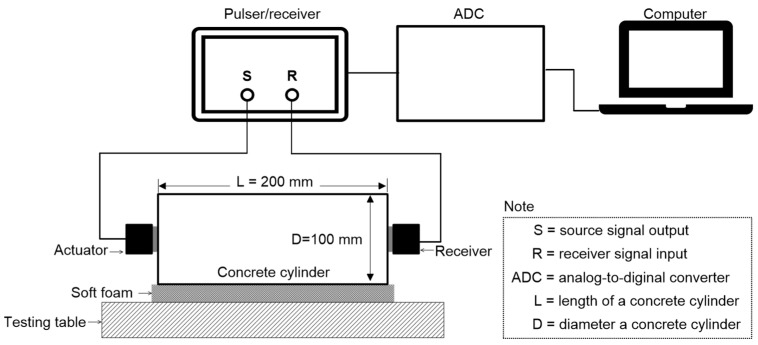
Test setup for ultrasonic pulse velocity measurements.

**Figure 3:**
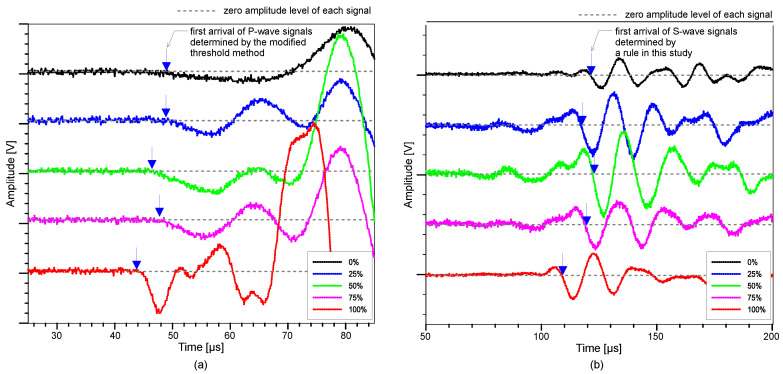
Typical time signals of ultrasonic pulse waves propagating through the MIX 1 concrete cylinders with various water saturation conditions: (**a**) P-wave and (**b**) S-wave signals.

A total of fifteen groups were assembled for this experiment. For each design mix, five specimens were prepared for the measurement from each saturation level, giving a total of 75 specimens. The standard test procedure according to ASTM C 597/C597M-16 was used to assess the P-wave velocity of concrete cylinders with five different saturation levels [75]. The study used a pair of transducers with about 50 kHz center frequency which can transmit and receive ultrasonic pulses (see Figure 2). Using a pulser-receiver (Panametrics 5077 PR, Tokyo, Japan), a 200 V square pulse with a duration of 10 μs was used to drive the source transducer (Olympus, Tokyo, Japan). The receiving sensor recorded transient stress waves that were created by the source sensor and propagated through the concrete. The received signal was digitized by a high-speed digital oscilloscope (NI-PXI 5101, Austin, TX, USA) with a total signal length of 0.001 s at a sampling rate of 10 MHz. The digitized data were transferred to a laptop computer for data storage and post-processing. Figure 3a presents the typical P-wave signals measured from the MIX 1 concrete cylinders used in this study with five different water saturation conditions (0%, 25%, 50%, 75% and 100%). The P-wave velocity of concrete, *V_p_*, was determined by dividing the travel distance by the travel time of the wave

(2) $$V_{P}=\frac{d}{\left(t_{a}-t_{d}\right)}$$

where *d* is the distance between transducers, *t_a_* is the time of first wave arrival and *t_d_* is the delay time, calculated during calibration of the probes. Delay time was determined when time for the first arrival wave was registered when the two transducers were positioned against each other. The first arrival time of the P-waves, *t_a_*, was determined by the modified threshold method [18].

The S-wave velocity of concrete was measured using the P-wave velocity method described in the previous section but using a different pair of transducers (40 kHz dry-point shear wave transducer produced by Proceq, Schwerzenbach, Switzerland). The S-wave transducer has a weight of 340 g with dimensions of 114 mm (length) by 84 mm (diameter), which is portable. Its eight dry point shear wave sensor array does not require extra coupling agent (such as a sticky and viscous coupling gel). This minimizes the influence of coupling conditions between the concrete surface and the transducer. Moreover, the shear wave sensors’ dry contact function substantially improves test speed while ensuring accurate and consistent data gathering. Figure 3b shows the typical impulse signals produced from the S-wave velocity measurement. The modified threshold technique was used to calculate the initial arrival time of the S-wave, similar to the P-wave velocity measurement method. However, precise detection of the first arrival time of the S-waves is often difficult due to the interference between direct P- and S-waves. Low amplitude P-wave components still appear in the time domain along with the S-wave components even when using S-wave transducers. For the present study, the first arrival of the S-waves was defined as the intersection of the fitting line to the first negative component of the S-wave and the calculated zero signal level, shown as a red dashed line in Figure 3b. To be clear, the initial low-amplitude signal was assumed to represent P-waves.

#### 2.1.3. Electrical Resistivity Measurement

**Figure 4:**
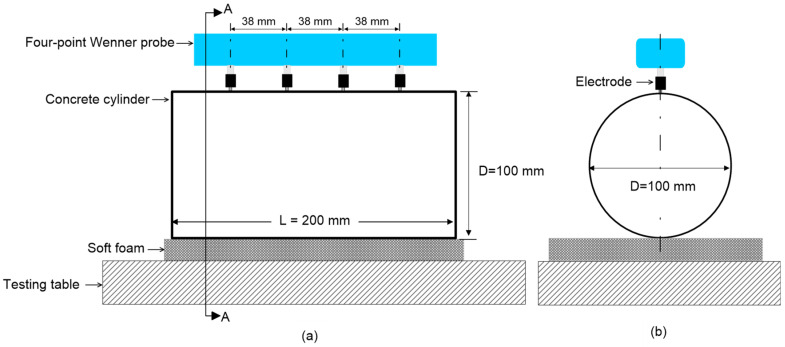
Test setup of electrical resistivity measurements of concrete: (**a**) four-point Wenner probe configuration, with an equal distance (=38 mm) between the electrodes, and (**b**) A-A section view of the Wenner probe configuration in Figure 4a.

Electrical resistivity (ER) of concrete was measured by a commercially available four-point Wenner probe with an electrode spacing of 38 mm. Four electrodes are aligned linearly at equidistant with each other (see Figure 4). The device follows the standard specification for AASHTO Designation T358-15 (surface resistivity indication of concrete’s ability to resist chloride ion penetration) [76]. Eight measurements were taken from each cylinder as prescribed from the specifications. The device shows an output value in kΩ-cm, which is the unit of measurement for apparent ER. Measurements were taken from five saturation conditions (0%, 25%, 50%, 75% and 100%).

#### 2.1.4. Measurement of Mechanical Properties

**Figure 5:**
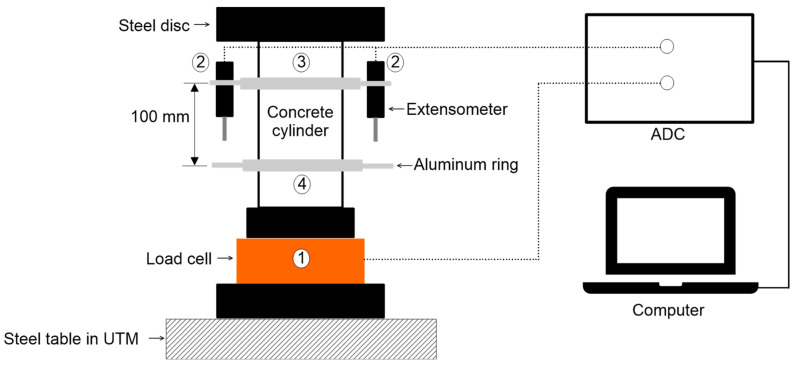
Test setup for the uniaxial compressive test for measurements of compressive strength and static modulus of elasticity of concrete cylinders.

After measuring the three NDE parameters (P- and S-wave velocities and ER), the compressive strength of the cylindrical specimens were measured using a 2000 kN universal testing machine (UTM, KST, Busan, South Korea). The specimens were at the age of around 226 to 263 days at the time of testing. The compressive strengths were tested in accordance with ASTM C39/C39M-20 [77] under displacement control. Uniaxial compressive testing was carried out at an axial movement rate of 2 mm/min. A load cell with a capacity of 2000 kN was used to measure the compressive loads applied to the surface of concrete cylinders, ① in Figure 5. Two sets of extensometers were mounted to two fixed frames to measure deformations, ② in Figure 5. It has two aluminum rings with screws for fastening the specimen, ③ and ④ in Figure 5. The screws from the top and bottom aluminum rings have a 100 mm spacing between them, which serves as a gauge length (L0) to determine axial strain from the evaluated deformations. A data acquisition device (DEWE43A, East Greenwich, RI, USA) with a sampling frequency of 100 Hz was used to digitize the load and deformation data obtained by the load cell and extensometers. 

### 2.2. Data Fusion

#### 2.2.1. Pre-Processing of Data

Before combining different parameters for estimation purposes, it is important to pre-process the data to be used for the different data fusion methods. Statistical analysis was used to verify the experimental data to be used for the study. Coefficient of variation was used to determine the variability of the NDT measurements as well as the compressive strength of the concrete specimens. The Pearson correlation was also calculated to determine the strength of association between the different parameters and to the dependent variable, compressive strength of concrete. Outliers were also determined to improve the estimation analysis. When there is an outlier, the corresponding parameter for that outlier data was also removed from the analysis. This is done so that there is a balanced amount of data for all the parameters used in the analysis.

#### 2.2.2. Multi-Variate Regression Analysis

A computer software (SPSS Statistics from IBM) was used for regression analysis and the nonlinear regression analysis were considered for evaluation. In this software, the nonlinear function model was defined by the user, and the coefficients and constant were estimated by doing many iterations until the differences between the previous and current estimates were minimal. Different functions were defined, depending on the number of combinations used, with different initial estimates for the coefficients and constants. The function defined is additive with each term characterized depending on the relationship of the compressive strength with a particular parameter. Previous studies have established that the relationship between wave velocities and compressive strength is exponential [18,24,78,79,80,81,82,83] and linear logarithmic between ER and compressive strength [32,33,34,84,85]. It is also well-known that there is linear relationship between density and compressive strength and inversely proportionality between water-to-cement ratio and compressive strength.

In general, the function that was defined in the program is presented below:

(3) $$\bar{f_{c}}=(B_{1}e^{A_{1}S})-(B_{2}e^{A_{2}P})+B_{3}D+B_{4}ln\left(ER\right)+\left(\frac{B_{5}}{B_{6}{}^{WB}}\right)-B_{7}$$

where $\bar{f_{c}}$ is the predicted compressive strength of the concrete, *S* is the S-wave velocity in km/s, *P* is P-wave velocity in km/s, *D* is the density in g/cm^3^, *ER* is the electrical resistivity in kΩcm and *WB* is the water-to-binder ratio. *B*_1_, *B*_2_, *B*_3_, *B*_4_, *B*_5_, *B*_6_, *B*_7_, *A*_1_ and *A*_2_ are the coefficients and constants estimated by the software. Depending on the number of parameters being combined, the number of the constants and the coefficients would change. In the equation presented, all five parameters were considered. Finally, the effectivity of the statistical method was compared to the results of the machine learning analysis.

#### 2.2.3. Artificial Neural Network (ANN)

**Figure 6:**
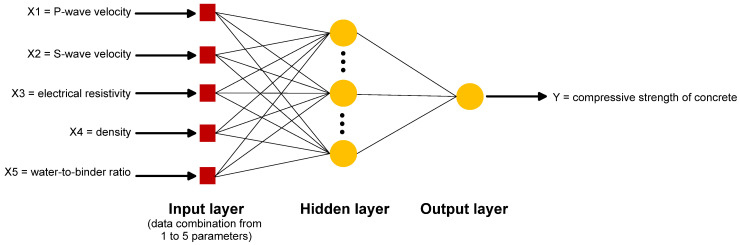
Schematic diagram for the ANN architecture used in analysis: this specific architecture considers the combination of five parameters for strength estimation.

For artificial neural network (ANN) method, one of the Machine Learning Toolbox from MATLAB software was used. The data set used for analysis was subdivided into three groups: training set, validation set and test set. Each group had a corresponding number of data that can be selected inside the toolbox. For the present study, the data distribution is as follows: 70% of data set for training, 15% for validation and 15% for testing. The analysis was done with one hidden layer selected for the ANN architecture as shown in Figure 6. For simplicity of the computation cost, the default setup from MATLAB (one hidden layer with eight neurons) was initially used for the analysis. After initial trainings and trials, one hidden layer with ten neurons was eventually selected since additional neurons added to the model do not significantly improve the performance of the model. Moreover, additional layers make the process more expensive in terms of storage and time while fewer neurons tend to underfit the experimental data. In implementing ANN, weights and biases are used to link all the neurons in each layer. Modifying the weights adjusts the values of the neuron from the preceding layers, which is then compensated for by the bias. The activation functions calculate the sum, which is then passed on to the next layer. This can be represented by the following equation:

(4) $$y_{i}=f\left(net\right)=f\left(\sum_{i=1}^{n}w_{ij}x_{i}+b_{j}\right)$$

where *y_i_* is the weighted sum in the *i*th neuron, *x_i_* is the input in the *i*th neuron, *w_ij_* is the weight between the *i*th and *j*th neurons, *b*_j_ is the bias in *j*th neuron and *f* is the activation function.

#### 2.2.4. Regression Learner (RL)

**Figure 7:**
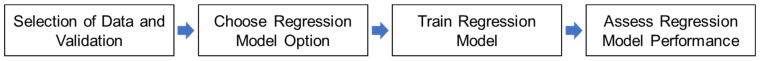
Methodology flowchart for using the regression learner application inside MATLAB.

Another one of the machine learning applications from MATLAB software was used, which is the regression learner (RL) method. For this study, only the models support vector machine (SVM) and Gaussian process regression (GPR) were considered after initial analysis with the other methods in MATLAB software. The general method used for regression training is shown in Figure 7.

Support vector machine (SVM) has been used widely for classification and recently, also been used for regression [15,16,17,18,19,20,21]. This method of regression was first established by Vladimir Vapnik [14]. In-depth discussion on how SVMs work has been discussed in previous studies [47,48,49,50,51,52]. For a training dataset of N points of the form (*x*, *y*) where *x* is the input vector, *y* is the target value and N is the size of the dataset acquired by the mapping of *x* into a high-dimensional feature space:

(5) f(x)=x′β+b

The goal of the SVM is to make the function as flat as possible, i.e., to minimize the structural risks in the model.

The nonparametric, Bayesian approach to regression known as Gaussian process regression (GPR) is making waves in the field of machine learning. GPR has many advantages, including the ability to work with small datasets and provide uncertainty measurements on forecasts. Because GPR is nonparametric (i.e., not constrained by a functional form), rather than computing the probability distribution of parameters of a single function, GPR computes the probability distribution of all admissible functions that fit the data. Few studies have already used GPR in estimating properties of concrete [59,60,61,62,63,64]. Similar to SVM, GPR are highly accurate but can be difficult to interpret.

## 3. Results and Discussion

### 3.1. Statistical Analysis of Experimental Data

It is important to analyze and verify the experimental data obtained from this study before doing the data combination. When investigating the consistency and reliability of the test methods used for this study, experimental variability was examined. For this research, the coefficient of variation (COV) was calculated and used as a means for evaluating the experimental variability of the NDT measurements as well as the compressive strength of the concrete cylinders. Moreover, outliers were determined by the quartile method. The summary of the statistical analysis is presented in Table 5.

The COV of the density ranges from 1.53% to 3.12% for concrete cylinders with different mixture proportions and saturation levels. The water-to-binder ratio was not included in the analysis as the data set was subdivided with respect to the different mix proportions. The COV for P-wave velocity ranges from 4.76% to 6.39% and for S-wave velocity, the range is from 1.43% to 2.17%. One outlier data was removed from the set of S-wave velocity before analysis. These values show that the P-wave velocity are more affected by the water saturation than the S-wave velocity, regardless of the type of liquid they were saturated with. For electrical resistivity (ER), the values of COV are very large because ER is greatly affected by the amount of water present in the concrete. The values from the oven-dried concrete specimens were not recorded since the values exceeded the capacity of the equipment. As for the compressive strength, the COV ranges from 11.55% to 20.22%. This can be explained by the levels of saturation that the concrete cylinders were exposed in. There were two outliers excluded from the analysis from this parameter.

Using the SPSS software from IBM, the different parameters were tested on their correlation with each other, as well as to the dependent variable, *f_c_*. Table 6 shows the Pearson correlation between the parameters. From the table, there is a strong positive correlation between the compressive strength and the S-wave velocity, followed by the density of the concrete. There is also a strong negative correlation between the compressive strength and water-to-binder ratio. Although there are small correlations between the other parameters and the compressive strength, the statistical analysis concluded that their correlations are still significant based on the calculated *p*-values.

Based on the calculated Pearson correlation coefficients, the most influential parameter that can affect the compressive strength is the S-wave velocity and the water-to-binder ratio. Analysis of this combination was also checked together with the electrical resistivity to consider the saturation degree and effect of the presence of NaCl in the environment. The combinations were chosen according to the correlation coefficients between parameters and the target variable, which is the compressive strength, and the practicality and ease of data collection, e.g., ease of use of NDT equipment. The combination of parameters considered for this study is listed as follows:

1. All five parameters: P, S, ER, D, WB.
2. P, WB, ER.
3. S, WB, ER.
4. P, WB.
5. S, WB.
6. P, ER.
7. S, ER.
8. P, S.
9. P.
10. S.

### 3.2. Multi-Variate Regression Analysis

**Figure 8:**
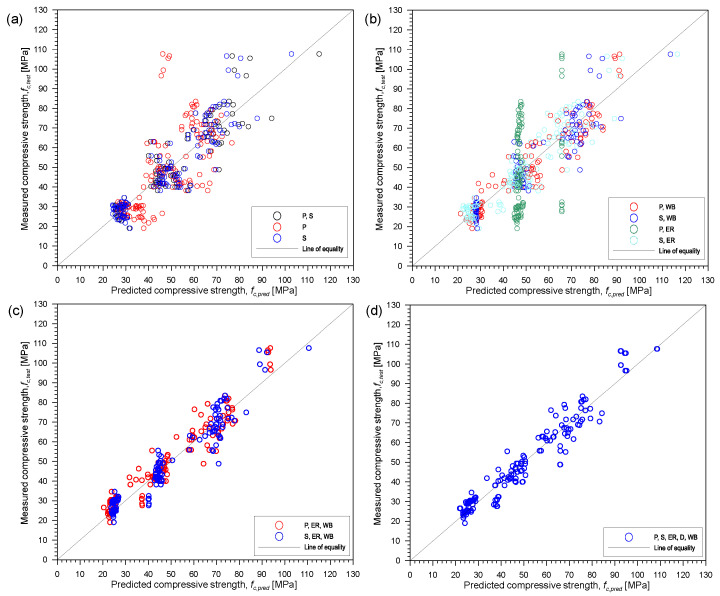
Correlation between actual *f_c,test_* and predicted *f_c,pred_* using different combinations of the five parameters from multivariate regression analysis: (**a**) only UPV parameters: P, S, or P and S, (**b**) combination of UPV and one other parameter: P and WB, S and WB, P and ER, or S and ER, (**c**) combination of UPV, ER and WB: P, ER and WB, or S, ER and WB and (**d**) five all parameters: P, S, ER, D and WB. Note—S: S-wave velocity, P: P-wave velocity, ER: electric resistivity, D: density and WB: water-to-binder ratio.

Figure 8 presents the correlation between actual and predicted compressive strength of concrete, *f_c,test_* and *f_c,pred_*, respectively, using different combinations of the five parameters (P-wave velocity, P; S-wave velocity, S; electrical resistivity, ER; density, D; water-to-binder ratio, WB) from multivariate regression analysis. Table 7 summarizes the resulting non-linear equations relating *f_c,test_* and *f_c,pred_*. For these equations, *f_c,pred_* is in MPa, P and S are in km/s, ER is in kΩcm and D is in g/cm^3^. As can be seen from Figure 8 and Table 7, all but two combinations of parameters gave acceptable values of coefficient of determination, R^2^, ranging from 0.818 to 0.930 with the exception of using P-wave velocity alone, with R^2^ of 0.440, and P-wave velocity and electrical resistivity with R^2^ of 0.118. Using ER alone was not included in the summary since from initial analysis, it gave an unacceptable of R^2^. This can be explained by the results obtained when the concrete specimens were under the oven-dried conditions and the high variability of the values across all saturation conditions. Using the combination of P-wave velocity and ER gave the lowest R^2^ of 0.118 among the 10 combinations considered. This can be explained by the effect of water saturation in both the P-wave velocity and ER values. On the other hand, using S-wave velocities alone already gave an acceptable value of coefficient of determination equal to 0.838.

It can be observed from Figure 8 and Table 7 that using a single parameter to estimate the compressive strength of concrete could still be improved by adding additional parameter. In the present study, among all the nonlinear equations, the combinations of the five parameters give the highest coefficient of determination equal to 0.93. Using only the S-wave velocity and water-to-binder ratio for estimating the compressive strength, the R^2^ value calculated by the nonlinear analysis is equal to 0.844. For the combination of P-wave velocity and water-to-binder ratio, the R^2^ value from the nonlinear analysis is equal to 0.886. As discussed from Section 1, studies have shown that combination of two or more parameters is better in estimating the compressive strength of concrete. Since several factors can affect each NDT parameter, additional parameters that can complement the other parameter would improve the performance of the regression model.

While regression analysis is easy to interpret, it entails a background in statistical training, and are frequently constrained by rigorous normality, variable independence, one-pass approximation, linearity, dimensionality, among others. In addition, it includes a lengthy and difficult computation and analytical technique since initial value must be assumed for each parameter. Background on the relationships and/or correlations of each estimator to the compressive strength must also be known to make the analysis and iterations faster.

### 3.3. Machine Learnig Methods

#### 3.3.1. Artificial Neural Network (ANN)

For this research, MATLAB was used with its integrated application of Neural Net Fitting. Seventy percent of the experimental data were allotted for training and fifteen percent each for validation and testing analysis. A few rounds of training were done with different configurations to determine the optimal model that can predict the compressive strength of concrete. The training algorithm used in this study is the Lavenberg–Marquardt (LM) network since it requires less time but still requires more memory. The same sets of combinations from the multi-variate regression were used for ANN analysis. ANN tool in MATLAB do not analyze single parameter for data fusion. For this analysis, the coefficient of determination was used as a criterion in determining the best combination of parameters in estimating the compressive strength of concrete. Table 8 presents the values of coefficient of determination, R^2^, of eight sets of combinations in compressive strength estimation.

**Figure 9:**
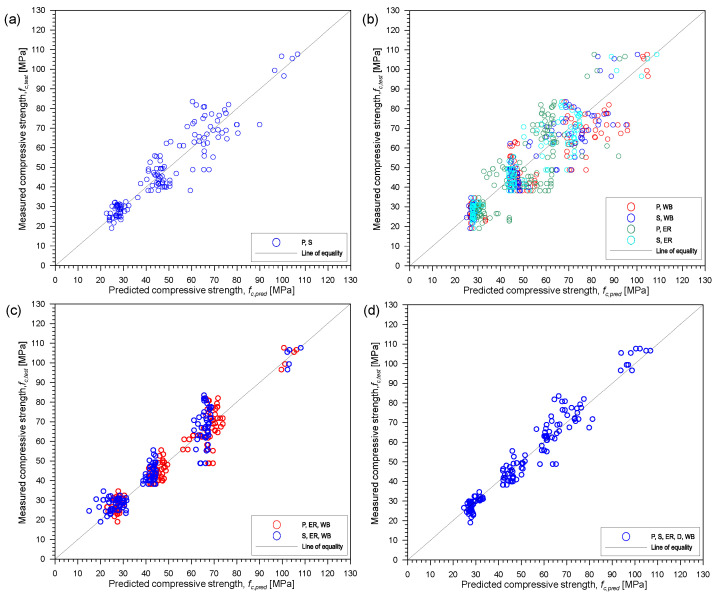
Correlation between actual *f_c,test_* and predicted *f_c,pred_* using different combinations of the five parameters from ANN analysis. (**a**) only UPV parameters: P, S, or P and S, (**b**) combination of UPV and one other parameter: P and WB, S and WB, P and ER or S and ER, (**c**) combination of UPV, ER and WB: P, ER and WB or S, ER and WB and (**d**) five all parameters: P, S, ER, D and WB. Note—S is S-wave velocity, P is P-wave velocity, ER is electric resistivity, D is density and WB is water-to-binder ratio.

Figure 9 presents the relationship between the actual or observed and predicted compressive strength of the concrete, *f_c,test_* and *f_c,pred_*, respectively, based on ANN analysis from the neural network fitting application in MATLAB. Predicted results from the toolbox can be also stored after the analysis to compare with the actual values. Based on the R^2^ values, the best combination from ANN analysis is the combination of five parameters. This is derived from ANN analysis with 10 hidden neurons. The ranking with respect to the best combination of parameters is the same from the multi-variate regression analysis. The R^2^ values from the different combinations are relatively close to each other. In this case, based on ANN analysis, all the combinations are sufficient to estimate compressive strength of concrete. Playing with different number of hidden layers of neurons, it was observed that increasing or decreasing the number would not greatly affect the overall R^2^ value of the combinations. It is better to use lesser number of hidden neurons since large number of neurons may result to overfitting the data. If that happens, it might mean that the model did not learn the trend and thus will not be able to generalize to new available data. While it is suggested to use fewer hidden layers for ANN, it is still best to be cautious because too few might yield to an underfitting or bias model that would not be able to fit new data as well.

**Figure 10:**
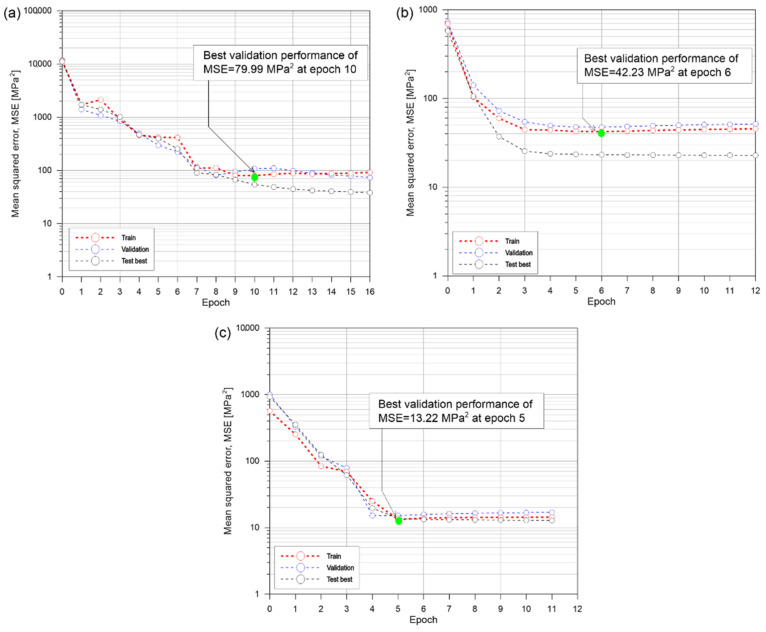
Sample parameter optimization using LM algorithm training: (**a**) 2 parameters, (**b**) 3 parameters and (**c**) 5 parameters.

As can be seen from Figure 10, the convergence of iterations became faster as the number of parameters combined increased. The values of mean squared error also decreased when the number of parameters was improved. The discrepancies in prediction between the validation and test data sets are also decreased, and the accuracy is greatly enhanced even with the fact that the prediction accuracy for the test and validation data sets is high due to concrete’s nonlinearity and to the restricted quantity of data.

From this analysis, although the combination of all five parameters gives the highest coefficient of determination, other combinations tested are sufficient to estimate the compressive strength of concrete. The practicality of gathering the parameters might be one criterion to determine which is the optimum combination to use in the estimation. Moreover, while ANN is considered more accurate than multi-variate regression analysis, the relative importance of the various parameters is not provided by the ANN. ANN also requires a large training data set to accurately predict a property. Moreover, the neural network being considered as a “black box”, its approximation will not provide any insight into the shape of the function. There is no straightforward relationship between the weights and the estimated function. Even determining which input feature is irrelevant is a challenge.

#### 3.3.2. Regression Learner: Support Vector Method (SVM) and Gaussian Process Regression (GPR)

**Figure 11:**
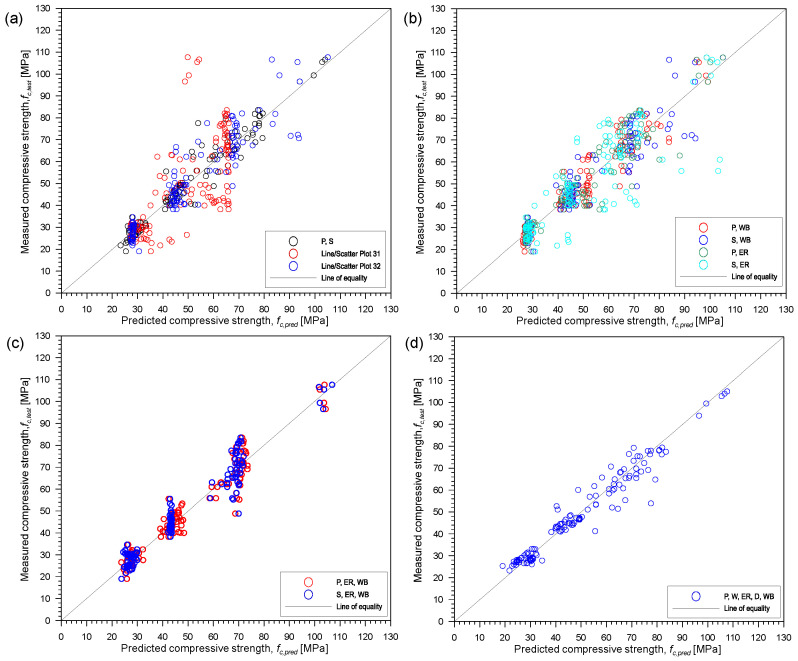
Correlation between actual fc,test and predicted fc,pred using different combinations of the five parameters from support vector machine analysis. (**a**) only UPV parameters: P, S, or P and S, (**b**) combination of UPV and one other parameter: P and WB, S and WB, P and ER or S and ER, (**c**) combination of UPV, ER and WB: P, ER and WB or S, ER and WB and (**d**) five all parameters: P, S, ER, D and WB. Note S is S-wave velocity, P is P-wave velocity, ER is electric resistivity, D is density and WB is water-to-binder ratio.

**Figure 12:**
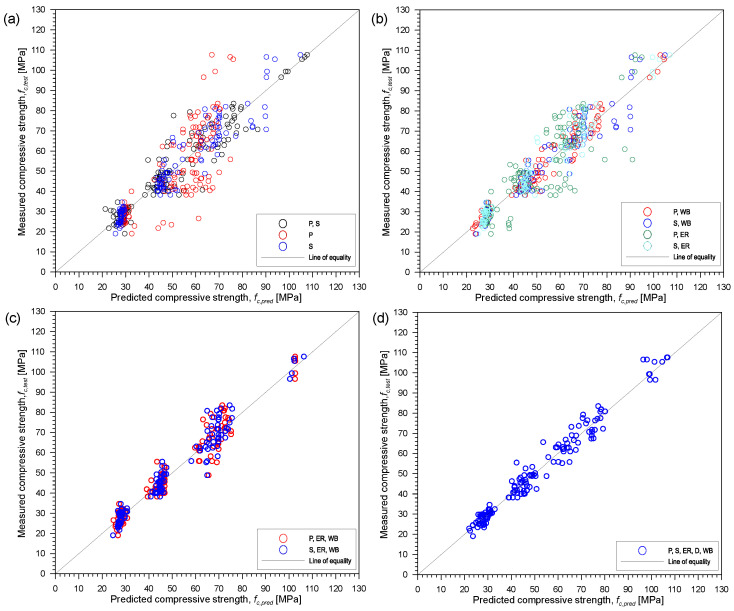
Correlation between actual *f_c,test_* and predicted *f_c,pred_* using different combinations of the five parameters from Gaussian process regression analysis. (**a**) only UPV parameters: P, S, or P and S, (**b**) combination of UPV and one other parameter: P and WB, S and WB, P and ER or S and ER, (**c**) combination of UPV, ER and WB: P, ER and WB or S, ER and WB and (**d**) five all parameters: P, S, ER, D and WB. Note S is S-wave velocity, P is P-wave velocity, ER is electric resistivity, D is density and WB is water-to-binder ratio.

Figure 11 and Figure 12 present the correlation between actual and predicted compressive strength of concrete, *f_c,test_* and *f_c,pred_*, respectively, using different combinations of the five parameters from SVM and GPR in the regression learner application in MATLAB. The correlation results from SVM and GPR are similar to those obtained from ANN in Figure 9. Consistent with the results from multi-variate regression and ANN, it can be confirmed that combination of two or more parameters can improve the accuracy of the predicted compressive strength of concrete. As can be seen from Figure 11a and Figure 12a, the arrangement of data points (presented with ‘o’ marks in red) is quite sparse for the estimation using only P-wave velocities indicating high variability of the estimated values compared to data points of the combination of five parameters (represented by ‘o’ marks in blue) shown in Figure 11d and Figure 12d for SVM and GPR, respectively.

Table 9 summarizes coefficient of determination (R^2^) values from SVM and GPR for the prediction of compressive strength of concrete using 10 different combinations of the five input parameters (P, S, ER, D and WB). Kernels and predefined models used in this study are also presented in Table 9. The suggested SVM model types differ for each combination being tested while GPR model types gave a consistent model type of exponential GPR. The R^2^ values from both methods are almost similar. Both methods gave the highest R^2^ to the combination of all five parameters while the lowest R^2^ came from using only P-wave velocities. The R^2^ values from SVM and GPR were comparable to the values from ANN if same input parameters are used.

### 3.4. Comparison of Methods and Parameter Combinations

The effectivity in estimating the compressive strength of the concrete using different methods were compared in terms of their R-squared. Table 10 presents the different R-squared values from different methods. As already discussed, ANN does not evaluate models with only one independent variable because ANN is for combination of two or more parameters to estimate or predict a certain characteristic.

The calculation of root mean square error (RMSE) is another way of comparing the different methods used for this study. The coefficient of determination, R-squared, is helpful when trying to rationalize what considerations might be driving the fundamental process of interest for the dependent variable. RMSE, on the other hand, gives an indication of how close the estimated values are to the actual observed data. This is useful in a range of applications to comprehend the accuracy of the model’s predictions. Table 9 presents the RMSE values of the different methods used for this study.

Based on the results of both the statistical criteria, R^2^ and RMSE, among the three methods from machine learning used, GPR is the most promising giving the good values for both R^2^ and RMSE. However, it should be noted that all four methods gave good values of R^2^ and RMSE as shown in Table 10 and Table 11. The discrepancies between the values are not significant. In this case, more study is needed on the use of GPR since research on this data fusion method is still limited.

Using only the single parameter, S-wave velocity, gives good values for the statistical criteria with values equal to 0.86 for coefficient of determination and 8.462 for RMSE. However, estimating concrete’s compressive strength using S-wave velocity needs more analysis and study since gathering of experimental and/or data is not easy and there is still limited research on this topic. On the other hand, the use of only P-wave velocity did not result in good values for the statistical criteria (R^2^ = 0.44 and RMSE = 18.04). This shows that P-wave velocity is greatly affected by the saturation condition of concrete. Adding another NDT parameter to both wave velocities may improve their performance. In this case, electrical resistivity, which is a parameter that is also easy to measure, was combined to the wave velocities. The results for both combination (P and ER, and S and ER) improved but not significantly for S-wave velocity. In terms of coefficient of determination, addition of ER to S-wave velocity improved its performance by only 6.98% but to P-wave velocity, the improvement was 70.45% except when the conventional regression analysis. Adding another property to the NDT parameters might improve the performance significantly. In this study, water-to-binder ratio, which is a concrete property available through the design documents, is added to the ultrasonic wave velocities. As can be seen from Table 10 and Table 11, the combinations of P- and S-wave velocities with both ER and WB significantly improved the performance from the combinations of only the ultrasonic wave velocities and ER. It can be observed this significant improvement especially for the P-wave velocity with more than 100% increase in its performance.

**Figure 13:**
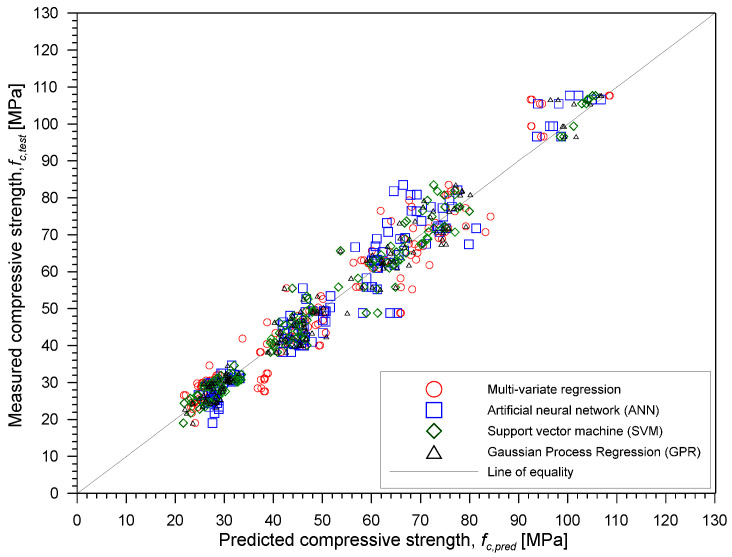
Correlation between measured and predicted compressive strength of concrete using the combination of five parameters from four different machine learning methods.

Based solely on the data analysis and on the values of R^2^, the best combination to estimate the compressive strength of concrete is the combination of all five parameters (P, S, ER, D and WB). The first three components are NDE parameters that can be measured in situ using the available NDT equipment while the last two components are generally available from the design documents. Among the four regression methods used, ANN gave the highest R-squared value equal to 0.97 while the use of GPR gave the lowest RMSE equal to 4.292. Figure 13 illustrates the comparison between the four methods using the best combination of parameters. Figure 13 presents the relationship between the actual or observed and predicted compressive strength of the concrete based on the combination of five parameters from all regression methods used in the study.

The analysis from RMSE calculations and an additional statistical parameter (mean absolute error or MAE from Table 12) gave a similar result as that of the coefficient of determination. The combination of all five parameters has the smallest RMSE and MAE among the different combinations tested from all methods of data fusion. One important observation is among the combinations tested for data fusion, combinations with ER parameter give the highest R-squared values and the lowest RMSEs and MAEs. This observation is sufficient to say that for concrete elements that are exposed to different levels of saturation and to the presence of chloride, ER together with other NDE parameters can give a more accurate estimation of the compressive strength of concrete.

## 4. Conclusions

This study aims to propose a combination of parameters to estimate the compressive strength of concrete exposed to different environmental conditions. The concrete samples used were cylindrical specimens from three different design mixtures. The inclusion of water-to-binder ratio was done to compensate for the different mixture proportions of the concrete specimens used in this study while the electrical resistivity is for the consideration of the presence of chloride in the water. Summarized below are two main findings from this study.

1. Based on the R-squared values and RMSE done for the study, using only one NDT parameter may not be sufficient to estimate the property of saturated concrete. Moreover, based on the same factors, the best combination of parameters in estimating the compressive strength of concrete is the inclusion of all five estimators used in this study—S-wave and P-wave velocities, electric resistant, density and water-to-binder ratio.
2. From all methods, artificial neural network showed the highest accuracy in terms of R-squared values while the Gaussian process regression gave the lowest value of root-mean-squared error.
3. Though combination of all parameters for compressive strength estimation of concrete gave the most accurate results, it is not always practical. From the point of view of practicality along with the results of the data analysis, the combination of three parameters—P-wave (or S-wave) velocity, electric resistivity and water-to-binder ratio—are sufficient to estimate the compressive strength of concrete when it is exposed to wet condition or marine environment. However, when choosing between P-wave and S-wave velocity measurement, it is more practical to use P-wave as it is easier to measure than the S-wave velocity.
4. This study also recommends to further investigate the potential use of S-wave velocity in estimating concrete under a saturated condition. This recommendation is based on the observations on the accuracy of using S-wave velocity, together with other parameters, in terms of the R-squared and RMSE values.
5. In the end, using only one NDT parameter is not enough in estimating the compressive strength of concrete under a saturated condition. Considering the practicality and ease of NDT measurement, the combination of P-wave (or S-wave) velocity, water-to-binder ratio and electrical resistivity might be good enough to estimate the compressive strength of concrete exposed in different saturation environments.

## Figures and Tables

**Table 1:** Summary of principles, advantages and limitations of popular data fusion methods in prior studies.

Data Fusion Method		Principles	Advantage	Limitations
Conventional Method	1. Regression Analysis	Conventional statistical approach in determining the relationship between a dependent variable and independent variable/s.	Allows researchers to look at relationships between variables in an overarching way and to quantify the relationship between variables.	Complex and involve high level mathematics that require a statistical program to analyze the data.
Machine Learning Method	2. Artificial Neural Network (ANN)	Computing technique designed to simulate the human brain’s method in problem-solving.	Information such is stored on the entire network, not on a database. Disappearance of a few pieces of information in one place does not prevent the network from functioning.	Require processors with parallel processing power, in accordance with their structure. For this reason, the realization of the equipment is dependent.
	3. Support Vector Machine (SVM)	Classifies the data using hyperplane which acts like a decision boundary between different classes.	Has good generalization capabilities which prevent it from over-fitting, and it can efficiently handle non-linear data using Kernel trick.	Algorithmic complexity and memory requirements are very high.
	4. Gaussian Process Regression (GPR)	Nonparametric, Gaussian process calculates the probability distribution over all admissible functions that fit the data set.	Can compute empirical confidence intervals and decide based on those if one should refit the prediction in some region of interest.	Use the whole samples/features information to perform the prediction so they are computationally expensive.

**Table 2:** Concrete mixture proportions of the concrete cylinders used in this study.

	Mixture Proportion (kg/m^3^)								
	W	C	S	G	SCMs		CA	W/B (%)	S_V_/A_V_
					FA	SC	AE		
MIX 1	170	99	884	931	33	198	1.98	51.52	0.490
MIX 2	170	110	858	923	37	220	2.57	35.43	0.495
MIX 3	160	312	725	876	63	250	6.88	25.60	0.456

Note—W: water, B: binder, S_V_: volume of sand, A_V_: volume of aggregates, W: water, C: Portland cement type I, S: sand, G: gravel, SCMs: supplementary cementitious materials, FA: fly ash type II, SC: blast furnace slag cement type II, CA: chemical admixtures and AE: high performance air-entraining agent.

**Table 3:** Summary of constant coefficients for the estimated saturation curves determined by non-linear regression analysis.

	$a_{1}$	$a_{2}$	$a_{3}$	$b_{1}$	$b_{2}$
MIX 1	9.985 × 10	4.312 × 10^4^	1.617 × 10^2^	5.509 × 10^2^	6.767 × 10^3^
MIX 2	9.987 × 10	2.245 × 10^4^	5.738 × 10	3.719 × 10^2^	4.788 × 10^3^
MIX 3	9.900 × 10	2.356 × 10^4^	1.314 × 10^2^	3.575 × 10^2^	4.902 × 10^3^

**Table 4:** Summary of approximate time t for the target saturation degree of specimens (25%, 50%, 75% and 100%) determined from the estimated saturation curves for the MIX 1, 2 and 3 concrete cylinders.

	Time t (min)			
	25%	50%	75%	100%
MIX 1	5.6	20.4	111.2	14,400
MIX 2	8.7	40.7	271.9	14,400
MIX 3	8.1	33.4	208.8	14,400

**Table 5:** Summary of statistical analysis of the parameters.

		D	S	P	ER	*f_c_*
Mix 1	N	50	50	50	40	50
	µ	2089.94	1967.07	3665.97	49.17	28.01
	σ	65.26	28.09	234.26	75.31	3.24
	COV	3.12	1.43	6.39	153.16	11.55
Mix 2	N	50	50	50	40	50
	µ	2266.98	2190.95	4268.88	46.60	47.80
	σ	46.62	47.54	246.37	47.51	7.88
	COV	2.06	2.17	5.77	101.96	16.48
Mix 3	N	50	49	50	40	48
	µ	2341.04	2325.08	4581.52	43.94	74.61
	σ	35.85	41.67	217.98	37.33	15.09
	COV	1.53	1.79	4.76	84.96	20.22

Note—D: density (g/cm^3^), S: S-wave velocity (m/s), P: P-wave velocity (m/s), ER: electrical resistivity (kΩcm), *f_c_*: compressive strength of concrete (MPa), N: number of samples, μ: average, σ: standard deviation and COV: coefficient of variable.

**Table 6:** Pearson correlation between the variables.

	WB	S	D	P	ER	SD	SAL	*f_c_*
WB	1	−0.964	−0.899	−0.854	0.000	0	0	−0.859
S	−0.964	1	0.86	0.8	0.073	−0.057	−0.007	0.874
D	−0.899	0.86	1	0.919	−0.25	0.378	0.041	0.648
P	−0.854	0.8	0.919	1	−0.308	0.386	−0.096	0.582
ER	0.000	0.073	−0.25	−0.308	1	−0.707	0	0.319
SD	0	0.378	−0.057	0.386	−0.707	1	0	0.022
SAL	0	0.041	−0.007	−0.096	0	0	1	−0.344
*f_c_*	−0.859	0.874	0.648	0.582	0.319	−0.344	0.022	1

Note—WB: water-to-cement ratio, S: S-wave velocity, D: density, P: P-wave velocity, ER: electrical resistivity, SD: degree of saturation, SAL: concentration of NaCl solution and *f_c_*: compressive strength of concrete.

**Table 7:** Summary of nonlinear equations obtained from different parameter combinations.

Combination	Equation	R^2^
P, S, WB, ER, D	$f_{c,pred}=\left(6.459\left(10^{-7}\right)e^{7.071S}\right)-\left(7.78\left(10^{-6}\right)e^{3.033P}\right)-19.106D+0.444\ln\left(ER\right)+\frac{288.725}{261.548^{WB}}+48.187$	0.930
P, WB, ER	$f_{c,pred}=\left(-2.366\left(10^{-5}\right)e^{2.846P}\right)+0.569\ln ER-\frac{414.979}{959.115^{WB}}+12.712$	0.920
S, WB, ER	$f_{c,pred}=\left(2.01\left(10^{-8}\right)e^{8.577S}\right)+0.681\ln ER-\frac{208.149}{282.163^{WB}}+11.123$	0.907
P, WB	$f_{c,pred}=\left(-0.006e^{1.824P}\right)-\frac{410.421}{391.294^{WB}}+114.346$	0.886
S, WB	$f_{c,pred}=\left(3.354\left(10^{-7}\right)e^{7.665S}\right)-\frac{131.526}{84.586^{WB}}+13.427$	0.844
P, ER	$f_{c,pred}=\left(1.4\left(10^{-8}\right)e^{-5.5P}\right)+0.860\ln ER+43.953$	0.118
S, ER	$f_{c,pred}=\left(0.10e^{2.806S}\right)+0.549\ln ER-0.939$	0.861
P, S	$f_{c,pred}=\left(0.011e^{3.694S}\right)-\left(1.421\left(10^{-8}\right)e^{-5.497P}\right)+12.479$	0.818
P	$f_{c,pred}=3.0876e^{0.6476P}$	0.440
S	$f_{c,pred}=0.1768e^{2.5728S}$	0.838

Note: D: density (g/cm^3^), S: S-wave velocity (km/s), P: P-wave velocity (km/s), ER: electrical resistivity (kΩcm) and $f_{c}$: predicted compressive strength of concrete (MPa).

**Table 8:** Coefficient of determination values (R-squared values, R^2^) of training results from artificial neural network (ANN) training for all experimental data.

Coefficient of Determination, R^2^				
Combination	Training	Validation	Test	Overall
S, P, WB, ER, D	0.98	0.98	0.98	0.97
P, WB, ER	0.97	0.98	0.96	0.96
S, WB, ER	0.95	0.97	0.95	0.94
P, WB	0.94	0.98	0.96	0.93
S, WB	0.91	0.93	0.93	0.89
P, ER	0.77	0.92	0.84	0.75
S, ER	0.94	0.93	0.92	0.92
S, P	0.93	0.95	0.94	0.92

**Table 9:** Coefficient of determination values (R-squared values, R^2^) of training results from regression learner training.

Coefficient of Determination, R^2^				
Combination	SVM		GPR	
	R^2^	Kernel	R^2^	Model
S, P, WB, ER, D	0.95	Quadratic	0.96	Exponential
P, WB, ER	0.94	Quadratic	0.94	Exponential
S, WB, ER	0.93	Quadratic	0.93	Exponential
P, WB	0.92	Medium Gaussian	0.92	Exponential
S, WB	0.85	Fine Gaussian	0.88	Exponential
P, ER	0.71	Fine Gaussian	0.70	Exponential
S, ER	0.86	Medium Gaussian	0.90	Exponential
S, P	0.88	Fine Gaussian	0.92	Exponential
P	0.35	Cubic	0.34	Exponential
S	0.84	Fine Gaussian	0.86	Exponential

**Table 10:** R-squared comparison between the different methods used in the study.

R-Squared				
Combination	ANN	SVM	GPR	Multi-Variate Regression
S, P, WB, ER, D	0.97	0.95	0.96	0.93
S, WB	0.89	0.83	0.85	0.84
S, WB, ER	0.94	0.93	0.93	0.91
P, WB	0.93	0.92	0.92	0.89
P, WB, ER	0.96	0.94	0.94	0.92
S	-	0.84	0.86	0.84
P	-	0.35	0.34	0.44
S, P	0.92	0.88	0.92	0.82
S, ER	0.92	0.86	0.90	0.86
P, ER	0.75	0.71	0.70	0.12

**Table 11:** Comparison of RMSE between the different methods used in the study.

RMSE (MPa)				
Combination	ANN	SVM	GPR	Multi-Variate Regression
S, P, WB, ER, D	4.988	4.971	4.292	5.879
S, WB	8.354	8.762	7.783	8.783
S, WB, ER	6.153	5.991	5.742	6.796
P, WB	9.476	6.476	6.163	7.522
P, WB, ER	5.493	5.485	5.305	6.4792
S	-	9.146	8.462	9.769
P	-	18.04	18.154	18.708
S, P	7.055	7.843	6.543	9.512
S, ER	6.745	8.2987	7.0455	8.293
P, ER	11.950	12.036	12.305	20.896

**Table 12:** Comparison of MAE between the different methods used in the study.

MAE (MPa)				
Combination	ANN	SVM	GPR	Multi-Variate Regression
S, P, WB, ER, D	3.579	3.568	3.058	4.4005
S, WB	6.291	6.230	5.741	6.297
S, WB, ER	4.551	4.374	4.240	5.190
P, WB	7.073	4.963	4.698	5.849
P, WB, ER	3.929	3.975	3.918	5.161
S	-	6.367	6.141	7.059
P	-	12.337	13.072	12.756
S, P	5.235	5.638	4.447	6.918
S, ER	5.005	6.035	5.121	6.577
P, ER	9.414	8.419	9.081	17.215

## Data Availability

Data are contained in this article. However, the data presented in this study are also available upon request from the corresponding author.

## Abbreviations

AAS	Activated alum sludge
ANN	Artificial neural network
COV	Coefficient of variation
*f_c_*	Compressive strength
D	Density
ER	Electrical resistivity
GPR	Gaussian process regression
GGBFS	Ground granulated ballast furnace slag
LM	Lavenberg-Marquardt
MAE	Mean absolute error
MSE	Mean squared error
NDE	Nondestructive evaluation
NDT	Nondestructive test
P	P-wave velocity
QA	Quality assurance
QC	Quality control
RL	Regression Learner
RMSE	Root-mean-squared error
SVM	Support vector machine
S	S-wave velocity
UPV	Ultrasonic pulse velocity
UTM	Universal testing machine
WB	Water-to-binder ratio
